# First Multi-Target Application of Exclusion Net in Nectarine Orchards: Effectiveness against Pests and Impact on Beneficial Arthropods, Postharvest Rots and Fruit Quality

**DOI:** 10.3390/insects12030210

**Published:** 2021-03-02

**Authors:** Valentina Candian, Marco Giuseppe Pansa, Karin Santoro, Davide Spadaro, Rossella Briano, Cristiana Peano, Luciana Tavella, Rosemarie Tedeschi

**Affiliations:** 1 Dipartimento di Scienze Agrarie, Forestali e Alimentari (DISAFA), University of Torino, Largo P. Braccini 2, 10095 Grugliasco, Italy; valentina.candian@unito.it (V.C.); marco.pansa@unito.it (M.G.P.); santorokarin@gmail.com (K.S.); davide.spadaro@unito.it (D.S.); rossellabriano@gmail.com (R.B.); cristiana.peano@unito.it (C.P.); luciana.tavella@unito.it (L.T.); 2 AGROINNOVA—Centre of Competence for the Innovation in the Agro-Environmental Sector, Largo P. Braccini 2, University of Torino, 10095 Grugliasco, Italy

**Keywords:** fruit moth, *Halyomopha halys*, fruit damage, *Monilinia fructicola*, predator, nutraceutical property

## Abstract

**Simple Summary:**

Recently, in fruit orchards, some well-established Integrated Pest Management (IPM) programs are losing their effectiveness and may be compromised by chemicals required to control new invasive pests. In this contest, exclusion nets represent an alternative sustainable control strategy. The use of a photoselective exclusion net was investigated in semi-field conditions as a tool to protect nectarine orchards against different pests in NW Italy. The presence and the abundance of key and new invasive pest populations, as well as the damage they caused on fruits, were evaluated. Moreover, any possible effect of the net on beneficial arthropods, postharvest rots and fruit quality and nutraceutical parameters were considered. The exclusion net significantly reduced pest populations allowing the production of healthier fruits due to a strong reduction of insecticide treatments. Moreover, no negative impact on postharvest rots, neither fruit quality nor nutraceutical properties were recorded.

**Abstract:**

Over the past few years, there has been an increasing interest in the development of alternative pest control strategies to reduce environmental impact. In this contest, exclusion nets have been evaluated as a sustainable alternative to pesticides. In this study, the use of a photoselective exclusion net was investigated in semi-field conditions as a potential strategy to protect nectarine orchards from different pests (i.e., fruit moths, *Halyomorpha halys* and *Drosophila suzukii*) in NW Italy. The presence and abundance of pest populations inside and outside the net, as well as the damage they caused on fruits, were evaluated. Moreover, any possible effects of the net on beneficial arthropods, postharvest rots and fruit quality and nutraceutical parameters were considered. The exclusion net significantly reduced pest populations. At harvest, fruit damage caused by *Grapholita molesta* and *H. halys* in netted plots was reduced up to 90% and to 78%, respectively, compared with insecticide-treated plots. The exclusion net allowed the production of healthier fruits with a strong reduction of insecticide treatments (up to seven less) and of their related costs without any negative impact on postharvest rots, neither fruit quality nor nutraceutical properties.

## 1. Introduction

In peach orchards, the management of pests of greatest concern such as *Grapholita molesta* (Busck) (Lepidoptera: Tortricidae), *Anarsia lineatella* Zeller (Lepidoptera: Gelechiidae) and *Myzus persicae* (Sulzer) (Hemiptera: Aphididae) have influenced the crop protection guidelines for years. In particular, in temperate regions, *G. molesta* is the main pest of stone fruits, and chemical control played a key role in its management; however, frequent insecticide treatments increased hazards for workers, consumers and the environment and often induced resistance in treated insect populations [1]. Resistance to 14 insecticides, including 10 organophosphates, was already developed in *G. molesta* in the ‘90s [2]. Therefore, alternative control methods with a reduction of the environmental impact compared with insecticides have been implemented in the last twenty years, mainly focusing on microbiological insecticides [3] and on mating disruption technique using synthetic sex pheromones [4]. Moth populations have been successfully controlled by the use of sex pheromones for several years; however, damage to shoot tips and fruit has started to increase in the last decade in some growing areas. In the last five years, infestations were observed in Italy (Emilia Romagna Region), probably due to the fragmentation of the orchards that increased border areas, negatively affecting the efficacy of this technique. Although in Italy the damage was not economically significant, the possibility of an increasing problem is of great concern [5]. Instead, in Australia, severe damage was recorded, in particular on the border of peach blocks treated with mating disruption technique adjacent to fruit blocks where insecticide treatments were used [6]. Furthermore, insecticide applications to supplement this strategy under high pest densities [7] and the chemical management required to control new invasive pests, such as the brown marmorated stink bug *Halyomorpha halys* (Stål) (Hemiptera: Pentatomidae), nullified the established Integrated Pest Management (IPM) programs making the mating disruption redundant and an additional cost. As an alternative, the application of exclusion nets to contain different pests reduces farmer reliance on agrochemicals, making it an environmental-friendly alternative to chemical pesticides [8] and a readily available tool for the management of pests not effectively controlled by insecticide treatments [9,10].

The use of the exclusion net was mainly investigated and applied in apple orchards to control damage by *Cydia pomonella* L. (Lepidoptera: Tortricidae) [11,12], aphids [13] and *H. halys* [14,15]. Furthermore, it has already been proven that the net interferes with *C. pomonella* biology [11,16,17], while for other pests, only the exclusion mechanism is known at the moment [18]. For other agroecosystems, research mainly focused on the influence of the net on fruit quality, photosynthesis, vegetative and reproductive growth, while implications on pest biology and exclusion mechanisms are still less investigated. To our knowledge, this is the first study assessing the exclusion net effectiveness in nectarine orchards in a multi-target approach. In our trials, different pest populations, fruit damage and the possible impact of the net on natural enemies, in particular predators, were monitored during the growing season and at harvest. Moreover, any possible effects on fruit quality as well as on postharvest diseases, mostly brown rots, were evaluated at harvest and after the storage period.

## 2. Materials and Methods

### 2.1. Experimental Sites

**Figure 1:**
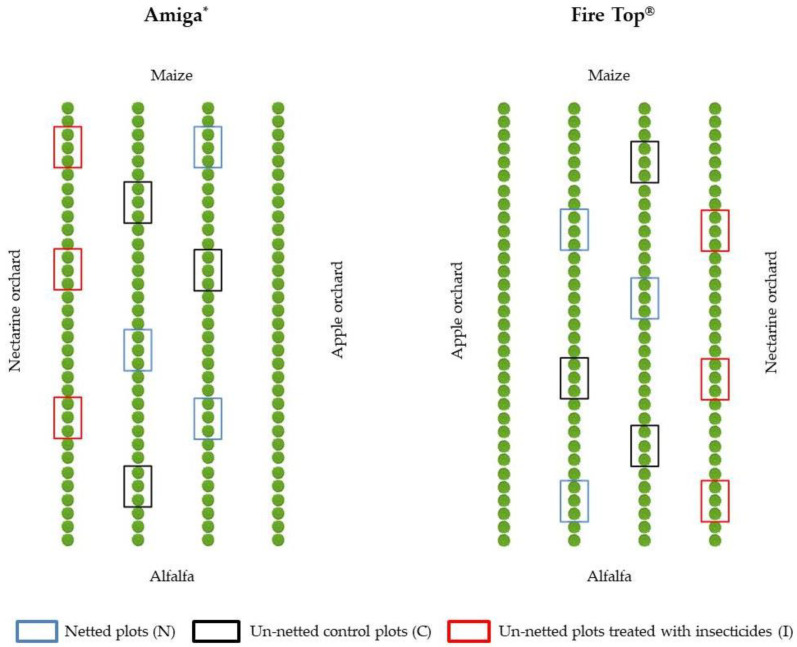
Experimental design in the two nectarine orchards (16 plants in each plot).

In 2016 and 2017, trials were carried out in two nectarine orchards (13 years old) located in Savigliano (Province of Cuneo, 44°37′19.5″ N, 7°37′32.6″ E, 321 m a.s.l., NW Italy) planted with the cultivars Amiga* (0.6 ha) and Fire Top^®^ (0.6 ha). In both orchards, the peach trees were arranged with a planting pattern of 1.2 × 4 m, and orchards were equipped with an anti-hail netting system. Trials were sorted in a randomized complete block design with three replicates for each of the following treatments: (i) netted plots (N), (ii) un-netted control plots (C), and (iii) un-netted plots treated with insecticides following the farmer’s schedule (I) (Figure 1). Un-netted insecticide-treated plots were included to evaluate the effectiveness of the net in comparison with the insecticide treatments in reducing pest fruit damage at harvest.

In each orchard, nine plots of 16 neighboring trees in a row were selected. The three netted plots were covered with the pearl anti-hail photoselective net Tenax Iridium (mesh 2.4 × 4.8 mm) (AGRINTECH S.r.l., Eboli (SA), Italy) set up by hooking the upper side to the anti-hail net support and fixing the lower side to the ground with metal pegs. The net was placed at the petal fall (17 May in 2016 and 21 April in 2017) and removed after harvesting. A knockdown treatment with the pyrethroid deltamethrin (Decis^®^ Jet, Bayer CropScience AG, Monheim am Rhein, Germany; 120 mL hL^−1^) was performed immediately after closing the net to eliminate pest populations. Later in the trials, no insecticides were applied in netted plots and un-netted control plots, while fungicides were applied in the same way in netted plots (directly through the net coverage) and in both un-netted plots, including control and insecticide-treated ones. The schedule for all pesticide treatments is reported in Table 1.

### 2.2. Pest and Beneficial Insect Monitoring

Pest populations, as well as beneficial arthropods, were monitored using traps and visual inspection every 10 days from the setting up of the nets until harvest. In particular, different pests, the peach moths *G. molesta* and *A. lineatella*, the brown marmorated stink bug *H. halys* and the fruit fly *Drosophila suzukii* (Matsumura) (Diptera: Drosophilidae) were specifically sampled.

#### 2.2.1. Fruit Moths

*Grapholita molesta* and *A. lineatella* were sampled using sticky delta traps baited with sex pheromones (CSALOMON^®^, Budapest, Hungary). In each orchard, a trap for *G. molesta* and another one for *A. lineatella* were placed in each netted (N) and un-netted control plot (C). Sex pheromones were replaced every four weeks to ensure their consistent effectiveness. Caught adults were transferred to the laboratory, where they were identified by morphological analysis of the aedeagus shape following dichotomous keys [19] and counted. Moreover, to evaluate any possible damage on shoots caused by *G. molesta* and *A*. *lineatella*, 30 shoots (10 shoots from three randomly selected trees) were checked in each N and C plot every 10 days from the setting-up of the nets until harvest.

#### 2.2.2. *Halyomorpha halys*

*Halyomorpha halys* was monitored through DEAD-INN™ Stink Bug Traps (AgBio, Westminster, CO, USA) (high 121.92 cm), baited with the Xtra Combo lure provided with the trap as already described in Candian et al. [14]. A trap was placed in a netted (N) and in an un-netted control plot (C) in both cultivars from mid-June until harvest in 2016, and only in the cultivar where the major number of catches and fruit damage was recorded during the first year of the trial (i.e., Fire Top^®^) from early May until harvest in 2017. The lure was changed every four weeks according to the manufacturer’s instructions. The specimens collected into the traps during each survey were identified and counted. Moreover, in each N and C plot, five branches of three randomly selected trees were shaken on a beating sheet (1 × 1 m) to assess the presence and the abundance of the pest during the growing season.

#### 2.2.3. *Drosophila suzukii*

Although *D. suzukii* is not a key pest in nectarine orchards, the abundance of this pest was monitored during the trials given its host potential, as reported by Bellamy et al. [20]. A trap filled with the feeding attractive Droskidrink (74.5% apple vinegar, 25% red wine and sugar) (Prantil, Priò di Vervò (TN), Italy) was hung in each netted (N) and un-netted control plot (C) at 1.50 m from the ground. It consisted of a 1.5 L transparent plastic bottle filled with 250 mL of Droskidrink and a drop of soap as a surfactant. The bottle was closed, and four holes were applied in its upper part in order to allow the insect entrance. At each survey, the collected *D. suzukii* adults were determined [21] and counted, and the attractive solution was replaced.

#### 2.2.4. Other Arthropods

The abundance of beneficial arthropods was evaluated thanks to sticky traps. A Glutor Yellow (25 × 20 cm) (BIOGARD^®^ Division, Cesena (FC), Italy) sticky trap was placed in each netted (N) and un-netted control plot (C) and changed every 10 days. The collected specimens were examined under a stereomicroscope for their identification, and at the same time, the predators were separated and counted.

### 2.3. Evaluation of Fruit Damage Caused by Pests

Samples of fruits were inspected visually throughout the growing season and at harvest to evaluate the damage caused by *G. molesta*, *A. lineatella* and *H. halys*. Following the setting up of the net, 30 fruits in each netted (N) and un-netted control plot (C) (10 fruits on three randomly selected trees) were checked every 10 days to evaluate the damage during the growing season. Overall, 180 and 240 nectarines were observed in each replicate in 2016 and in 2017, respectively.

At harvest, 480 nectarines were picked in each N, C, and I plot per year, with a total of 8640 fruits harvested in a peach orchard in the two years. The number of fruits damaged by *G. molesta* and *H. halys* was recorded. According to Acebes-Doria et al. [22], nectarines were considered damaged by *H. halys* if punctures, dimples, gummosis, fruit deformations, areas with superficial discoloration with or without depressions and areas with necrotic tissue after slicing the fruits were observed.

### 2.4. Evaluation of Postharvest Rots

In both years, samples of harvested nectarines were selected to evaluate the incidence of postharvest diseases after the storage period. For each treatment (N, C, I), 900 nectarines were collected in plastic boxes (50 fruits per box). Nectarines were stored in a normal atmosphere (0 °C, 90% RH) for 7 days. The incidence of postharvest rots was evaluated at harvest, after storage and after further 7 days of shelf life at 20 °C. Fungal isolation was performed from fruit showing disease symptoms. Pathogens were isolated by transferring small pieces of symptomatic fruit tissues, previously washed in 1% sodium hypochlorite and rinsed in sterile deionized water, onto potato dextrose agar (PDA, Merck, Darmstadt, Germany) plates amended with 25 mg L^−1^ streptomycin sulfate (Merck). A 7-day-old culture was used for observation of the fungal structures under an optical microscope. For *Monilinia* spp. isolates, molecular identification was performed by extracting DNA according to Franco Ortega et al. [23] and multiplex PCR with specific primers, according to Côté et al. [24].

### 2.5. Fruit Quality and Nutraceutical Analyses

Fruits were analyzed for the color index, firmness, total soluble solids and nutraceutical parameters (total anthocyanins and total polyphenols) at harvest following protocols already described in Candian et al. [14]. Since the results obtained in 2016 were previously reported in Candian et al. [14], only the ones recorded in 2017 will be shown. In 2017, for each treatment (N, C, I) and for each orchard, 80 fruits were analyzed for the color index, firmness and total soluble solids. For the analysis of the total anthocyanin and the total phenol, the skin and the fruit pulp were mixed. Every sample came from 18 fruits randomly selected per treatment and orchard.

### 2.6. Statistical Analyses

The statistical analyses were performed using SPSS v24.0 (SPSS Inc., Chicago, IL, USA), and outcomes were considered significant at *p* < 0.05. The numbers of *G. molesta*, *D. suzukii* and predators collected with traps were compared using a *t-*test for two independent samples. The percentages of fruits damaged by *G. molesta* and *H. halys* at harvest were compared using a generalized linear mixed model (GLMM; random effect: plot; fixed effects: treatment, block) with a binary distribution and logit link and Bonferroni correction. Block effect was used in order to assess if pests were more concentrated on the borders or in the middle of the orchards. Data on postharvest rots, quality and nutraceutical parameters of fruit at harvest were checked for homogeneity of variance (Levene test) and normality (Shapiro–Wilk test) and compared using one-way analysys of variance (ANOVA). In case of significant differences, means were separated by Tukey’s test. If the assumptions of ANOVA were not met, data were compared using the Kruskal–Wallis test, and means were separated with the Mann–Whitney U test.

## 3. Results

### 3.1. Monitoring of and Damage Caused by *Grapholita molesta* and *Anarsia lineatella*

*Grapholita molesta* and *A. lineatella* were collected with pheromone traps in both years. *Anarsia lineatella* was never found under the net, while a few specimens were collected in un-netted control plots (C). In 2016, only one specimen was observed in both cultivars, while in 2017, four specimens were sampled in Fire Top^®^. No shoots and fruit damaged by this pest were observed in our trials. *Grapholita molesta* was collected in both netted (N) and un-netted control plots (C) with pheromone traps. It was detected under the exclusion net with average weekly catches exceeding 10 specimens (the threshold for the application of an insecticidal treatment in IPM program in NW Italy) only in 2017 and only the week before harvest (Amiga*: 28 specimens, Fire Top^®^: 26 specimens). Significant differences between the treatments were observed in 2016 (t-test = Amiga*: df = 4, t = 6.63, *p* = 0.003; Fire top^®^: df = 4, t = 7.78, *p* = 0.001) and in 2017 (t-test = Amiga*: df = 4, t = 8.27; *p* = 0.001; Fire Top^®^: df = 4, t = 4.42, *p* = 0.012) with higher catches always recorded in C (Table 2).

The damage observed on shoots during the growing season was truly irrelevant: only three damaged shoots were observed in C and in N, respectively, in 2016. No fruits damaged by *G. molesta* were observed by visual inspections until 10 days before harvest. However, at harvest, damaged fruits were recorded in all three treatments (N, C, I), although in an uneven pattern. In 2016, the damage caused by *G. molesta* was negligible in both orchards. Even though significant differences between the treatments were not found, lower damage (in Amiga*) or even the absence of damage (in Fire Top^®^) was recorded in N. In 2017, significant differences between the treatments were observed both in Amiga* (GLMM: df = 2, 10; F = 28.85, *p* = 0.000) and in Fire Top^®^ (GLMM: df = 2, 10; F = 12.72, *p* = 0.000), with significantly lower damage in N (Table 2). Moreover, the GLMM was used to analyze the block effect in order to assess if the pest was more concentrated on the borders or in the middle of the orchards. Significant differences were recorded only in Amiga* in 2016 (GLMM: df = 2, 10; F = 9.58, *p* = 0.005), with higher damage in the edge bordering alfalfa compared to the orchard center.

### 3.2. Monitoring of and Damage Caused by *Halyomorpha halys*

This pest was captured by traps only during the first year of the trial in the un-netted control plot (C) (1 nymph and 1 adult in Amiga* and 8 nymphs and 1 adult in Fire Top^®^) as reported in Candian et al. [14] while, using the beating sheet, only one *H. halys* adult was collected in 2017 in one un-netted control plot (C) (Fire Top^®^) in June. Moreover, as already observed in the first year of the trial, during the growing season, a low number of damaged fruits was recorded by visual inspection in all the orchards in 2017. Out of 240 checked fruits, no damaged fruits in N and six damaged fruits in C (0.83%) were observed in Amiga* while six damaged fruits (0.83%) and 46 (6.39%) were observed in Fire Top^®^ in N and in C, respectively.

**Figure 2:**
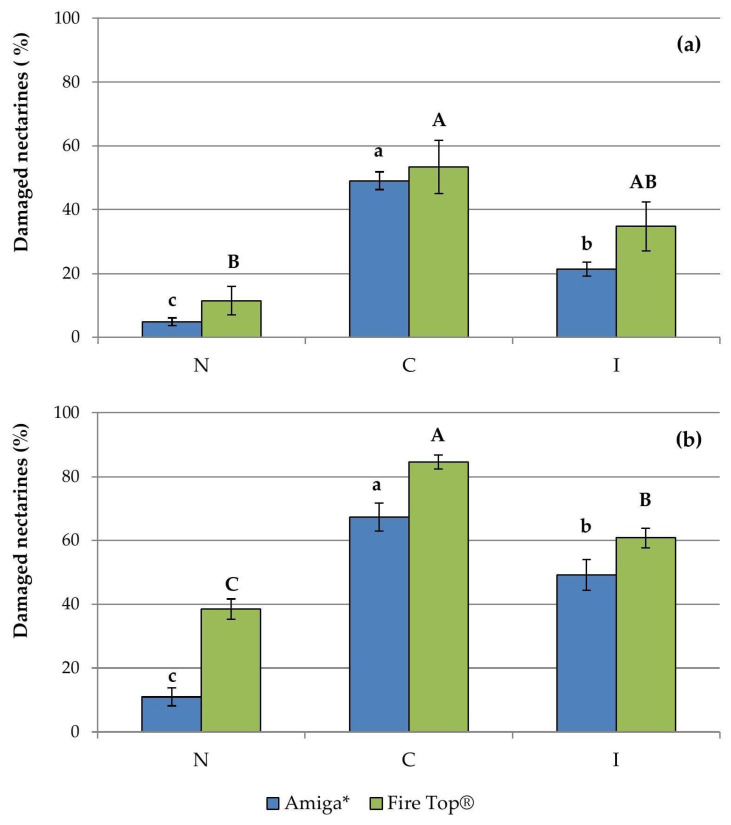
Percentages of nectarines damaged by *Halyomorpha halys* (mean ± SE) on fruits sampled at harvest in 2016 (**a**) and in 2017 (**b**) (no. = 1140 fruits per treatment per year) (N = netted plots, C = un-netted control plots, I = un-netted plots treated with insecticides). For each cultivar, histograms with different letters are significantly different by the GLMM analysis (Bonferroni correction, *p* < 0.05).

The damage on fruits observed in each orchard at harvest is reported in Figure 2. In both orchards and in both years, the trees in N and C plots where the pheromone trap was placed (i.e., one of three plots per treatment and orchard) showed the highest fruit damage rate in the respective treatment. Significant differences between the three treatments (N, C, I) were observed both in 2016 (GLMM= Amiga*: df = 2, 10; F = 65.88, *p* = 0.024; Fire Top^®^: df = 2, 10; F = 7.74, *p* = 0.009) and in 2017 (GLMM= Amiga*: df = 2, 10; F = 32.96, *p* = 0.000; Fire Top^®^: df = 2, 10; F = 50.87, *p* = 0.000), with a significantly lower damage in N. Significant differences between the blocks were recorded only in Amiga* in 2016 with a higher concentration on the borders as reported in Candian et al. [14].

### 3.3. Monitoring of *Drosophila suzukii*

The first specimens were collected in mid-June in 2016 and in early May in 2017. During the first year, in Amiga*, 21 and 182 *D. suzukii* were totally collected in netted (N) and un-netted control plots (C), respectively, while in Fire Top^®^ 39 and 387 specimens were trapped in N and C, respectively. Significant differences between the treatments were recorded both in Amiga* (t-test: df = 4, t = 15.21, *p* = 0.000) and in Fire Top^®^ (t-test: df = 4, t = 4.41, *p* = 0.012) (Table 3). In 2017, catches were slightly lower than the previous year, with 21 specimens collected in N and 64 in C in Amiga* and 36 specimens trapped in N and 101 in C in Fire Top^®^. Significant differences between the treatments were observed both in Amiga* (t-test: df = 4, t = 4.93, *p* = 0.008) and in Fire Top^®^ (t-test: df = 4, t = 3.17, *p* = 0.034) (Table 3).

### 3.4. Monitoring of Beneficial Insects

**Figure 3:**
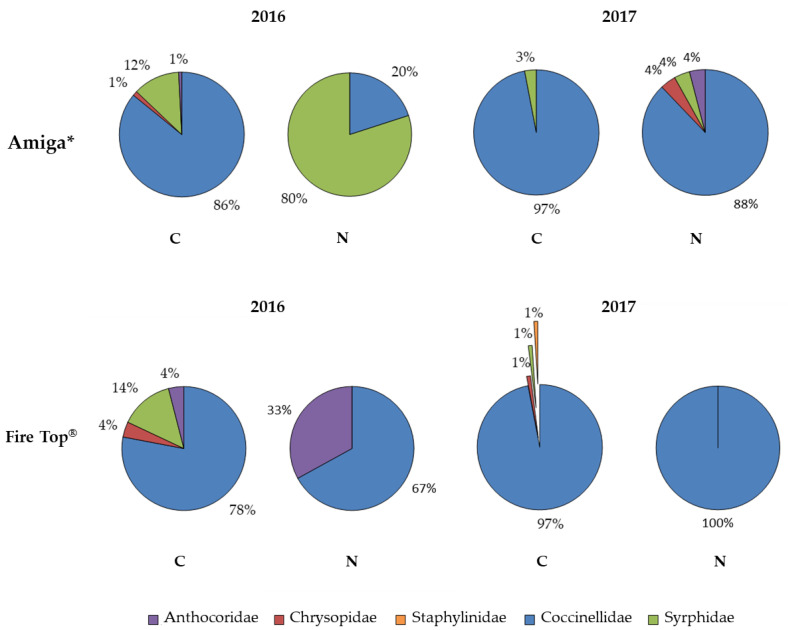
Percentages of specimens belonging to Anthocoridae, Chrysopidae, Staphylinidae, Coccinellidae and Syrphidae collected with yellow sticky traps (N = netted plots, C = un-netted control plots).

In both orchards, specimens belonging to Anthocoridae, Chrysopidae, Staphylinidae, Coccinellidae and Syrphidae collected with yellow sticky traps were grouped and statistically analyzed. Significant differences between the treatments were observed both in 2016 (t-test = Amiga*: df = 4, t = 12.12, *p* = 0.000; Fire Top^®^: df = 4, t = 13.99, *p* = 0.000) and in 2017 (t-test = Amiga*: df = 4, t = 23.29, *p* = 0.000; Fire Top^®^: df = 4, t = 5.99, *p* = 0.004) (Table 3) with higher captures in un-netted controls (C). The percentages of specimens collected by traps and belonging to the different families are reported for each treatment and year in Figure 3.

### 3.5. Postharvest Rots

At harvest, nectarines from both cultivars and both years did not show any rot (data not shown). After storage for 7 days at 0 °C, no significant differences were observed between the three treatments (N, C, I) in 2016 and in 2017, except for the nectarines Fire Top^®^ harvested in 2017 (ANOVA: df = 2, 6; F = 8.67, *p* = 0.017). In this case, fruits from un-netted plots treated with insecticides (I) showed a significantly higher incidence of brown rots at harvest (Table 4). After shelf life at 20 °C, significant differences between the three treatments were observed for postharvest rots on nectarines both in 2016 (one-way ANOVA= Amiga*: df = 2, F = 9.51, *p* = 0.005; Fire Top^®^: df = 2, F = 3.96, *p* = 0.037) and in 2017 (one-way ANOVA = Amiga*: df = 2, F = 8.49, *p* = 0.018; Fire Top^®^: df = 2, F = 4.53, *p* = 0.046) with lowest incidence of postharvest rots after shelf life recorded in netted plots (N) (Table 4). A significantly higher incidence of postharvest rots in shelf life was recorded in C compared to N. No differences were observed between I and N, except for nectarines Fire Top^®^ harvested in 2016. Most rots were brown rots, caused by *Monilinia fructicola*. Only in nectarines Fire Top^®^ harvested in 2017, a low percentage (14%) of postharvest rots was caused by *Rhizopus stolonifera*, while the rest was due to *M. fructicola*.

### 3.6. Fruit Quality and Nutraceutical Analyses

Significant differences between the three treatments (N, C, I) were observed for the fruit quality only in 2017. The highest values for the total soluble solids in Amiga* (one-way ANOVA: df = 2, 227; F = 4.58, *p* = 0.011), and for the color index (one-way ANOVA: df = 2, 227; F = 11.09, *p* = 0.000) and the total solid soluble (Kruskal–Wallis test: df = 2, χ^2^ = 31.28, *p* = 0.000) in Fire Top^®^ were recorded in C and I plots (Table 5). No significant differences were found between the three treatments for the anthocyanin and polyphenol content. However, a higher concentration of total polyphenols was observed in N plots in both cultivars (Table 5).

## 4. Discussion

In the last years, the use of the nets in stone fruit orchards was mainly evaluated as an anti-hail device [10,18] in relation to the qualitative and quantitative impact on fruit production [25,26,27,28,29,30,31,32] and/or in relation to plant physiology [26,31]. This study deals with the application of the net in a multidisciplinary approach focusing on pest and disease management and on qualitative–quantitative aspects in fruit production. Recently, indeed, the efficacy of well-established IPM procedures against the nectarine key pest *G. molesta* started to fail. This pest has been successfully controlled by the mating disruption for several years; however, in small and fragmented peach and nectarine orchards, significant fruit damage was recorded under this pest control strategy [5,6]. Therefore, in the perspective of a multidisciplinary and multi-target approach, it is necessary to investigate the exclusion net effectiveness also against this key pest. The exclusion net used in our trials proved to be a promising exclusion system that can prevent the attack of more than one insect pest at a time without any impact on the occurrence of some physiological disorders and diseases and preserving the production of healthier fruits with a strong reduction of insecticide treatments (in our trial, up to seven fewer) and their associated costs. It should be taken into account that in our trials, the exclusion net was applied in small plots and not in a row-by-row or in a single plot exclusion-net system as in a real field situation. However, the promising results give good prospects for a successful large-scale field application in commercial orchards.

In our trials, the pearl anti-hail photoselective net drastically reduced both *G. molesta* and *H. halys* populations and their damage on fruit in comparison with insecticide-treated plots. The exclusion net not only acts as a physical barrier against *G. molesta* but also may interfere with its biology. Although for *G. molesta,* this aspect has not yet been investigated in detail and it was not the purpose of our research, it is reasonable to assume that delay in adult development, difficulty in locate calling females, interference of the approaching phase in courtship and mating as well as visual disturbances of the searching males may occur under the net coverage as already proved for *C. pomonella* [11,17]. Catches under the exclusion net may be due to specimens that have passed through the net or developed from eggs laid through the net mesh. Moreover, it is important to note that our experimental condition was extreme since the pheromone trap for the pest monitoring was set up under the net coverage and may have strongly attracted the specimens under the net, a situation that would not occur in commercial orchards. Under the exclusion net, the damage on fruits caused by *G*. *molesta* was reduced up to 90% compared with insecticidal treatments.

As we already reported in Candian et al. [14], catches of *H. halys* obtained with the monitoring techniques used did not reflect the real abundance of the pest in the field. In the light of recent evidence [33], the low attractiveness obtained in the present study is more reasonably due to the inefficacy of the used lure formulation rather than to *H. haly*s haplotypes present in our area as previously supposed in Candian et al. [14]. Moreover, a greater attractiveness of kairomones emitted by plants could affect the pest response to the pheromone. In fact, the area of arrestment may be larger for the plant volatiles compared with the aggregation pheromone increasing the possibility that *H. halys* remains outside the pheromone trap capture range [34]. In our trials, even though catches were almost nil or very low, the best evidence for the effectiveness of the net against this pest comes from the assessment of damaged fruits at harvest. In netted plots, the damage was reduced up to 78% compared with insecticidal treatments. Only in Fire Top^®^ in 2017, higher damage (up to 38.5%), compared with the one recorded in the previous year, was observed in netted plots although still significantly lower than in un-netted plots (C, I). The higher pressure of this pest in the field, compared with the one recorded in the first year of the trials, and the contact of the net with the vegetation due to the great vegetative growth rate of the cultivar may have influenced the net physical barrier proprieties. Moreover, Chouinard et al. [18] reported that the second-stage nymphs could pass through nets with a mesh even smaller than the one we used (2.2 × 2.2 mm); therefore, juvenile stages could have passed the net and damaged the fruits. Significant differences for the block effect were recorded only in Amiga* in 2016, but in general, the damage was higher on netted and un-netted trees closer to the orchard edges, as already observed by Leskey et al. [35].

Although the main objective of this research was not to investigate the net effectiveness specifically on *D. suzukii*, this pest population was significantly reduced under the net, probably due to the optical properties of the used photoselective net as we already observed in apple orchards [15]. At the same time, the pearl net could affect aphid infestations [36] even if, in our trials, aphids were never observed, probably due to the late start of monitoring. The influence of the exclusion nets on aphid populations is still unclear and is largely influenced by the net effects on their predators and parasitoids [10,13]. It has been reported that using photoselective nets, the optical disruption caused by the reflected light may interfere with distant host finding by the pests and the light inside the net, containing less UV light or higher levels of reflected/scattered sunlight, could deter the pest landing [37]. This light disruption may affect beneficial insects too. Possible effects on predators were evaluated all along the trials with yellow sticky traps. Generally, the net mesh was large enough to allow tiny beneficial insects to pass through (mainly *Stetorus* spp. (Coccinellidae), Anthocoridae and Staphylinidae). Larger-size insects such as Chrysopidae and Syrphidae were collected only in the first weeks after the setting up of the net, probably for the presence of some individuals (e.g., eggs and/or juvenile stages) on the plants before the net installation. Predators belonging to the different families (mainly Coccinellidae) were recorded in different percentages in N and C plots, although in an uneven pattern. In agreement with Dib et al. [13] and Romet et al. [38], a significantly lower number of predators was collected under the net in all the orchards. However, during the first year of the trials, the predator abundance was also evaluated after the harvest with a knockdown treatment (pyrethroid deltamethrin), and it was not negatively influenced by the net [14]. It is probable that the yellow sticky traps lose their attractiveness under photoselective nets or the light modification altered both flight activity and behavior of beneficial insects, as already observed for some insects [37,39,40,41].

The mesh size is a critical issue for not only the exclusion effectiveness but also regarding its influence on the microclimate under the net, the incidence of diseases and the fruit quality and yield. In our trials, the temperature and relative humidity inside and outside the net coverage was recorded, but no differences were observed (data not shown). The lack of differences in microclimatic conditions did not favor the development of fungal pathogens in fruits from netted plots, as already demonstrated in other experiences performed with nets on stone fruit [42,43]. No differences among the treatments (N, C, I) were observed on the incidence of postharvest rots at harvest or after storage, except for the trial performed on Fire Top^®^ in 2017. In contrast, significant differences could be observed on both cultivars and years after shelf life: in fruits coming from netted plots, the rot incidence was always lower than in fruit from un-netted plots and even lower than in fruits from un-netted plots treated with insecticides, as previously shown on peach by Salvador and Fideghelli [44]. This latter information could greatly favor the development of anti-insect nets as an alternative treatment not only for insect control but also for the reduction of postharvest rots [45]. The lower rot incidence on fruits from netted plots could be related to the lower number of damage and wounds created by insects on the fruits. Insect wounds could represent one of the main entrances for postharvest rots [46]. Several authors already reported the positive effects of photoselective nets on stone fruit quality [25,27,30]. In our trial, significant differences between the treatments (N, C, I) were observed at harvest in 2017 but not in 2016 [14]. These differences, even if minimal, are probably due more to the timing of harvest than to the net influence. Nutraceutical parameters were never negatively influenced by the net coverage.

Nowadays, climate change and global warming are the most pressing issues. Shifting in global temperature and humidity is producing rapid evolutionary changes in many animal species, including agricultural pests and disease vectors [47], also influencing insect migration. Climate-induced early emergence, altered distribution and shifting temporal abundance on a large number of insects have already been confirmed [48,49,50,51,52,53,54]. In this scenario, the perspectives and effectiveness of the use of exclusion nets in fruit orchards could be enhanced. Currently, considering the particular attention to safeguarding the environment, one of the main concerns surrounding the application of plastic netting regards their end-of-life disposal. However, the use of biopolymers is a potential alternative that combines the benefits of two eco-friendly technologies (biopolymers and exclusion netting) to create a more sustainable alternative to pesticides [55].

## 5. Conclusions

The obtained results refer to a specific geographical area (NW Italy) and pest population densities; however, they are a useful starting point for applying them also on a large-scale (e.g., row-by-row, single plot exclusion-net) and in other areas characterized by different climatic conditions. In our trials, the pearl photoselective net proved to be effective in controlling more than one pest species at a time and the damage they cause to fruit, not only for the exclusion function but also for the optical effects, representing a very promising and sustainable tool for IPM orchards. In parallel, this net allowed the production of healthier fruits due to a strong reduction of insecticide treatments and of their related costs without any negative impacts on postharvest rots, neither on fruit quality nor on nutraceutical properties. In the context of global warming, which favors the increasing occurrence of exotic pests, the presence of exclusion nets can represent a “ready-to-use” tool against new invasive pests.

## Figures and Tables

**Table 1:** Insecticidal and fungicidal treatments applied in both orchards from the net setting-up until harvest in 2016 and 2017 (N = netted plots, C = un-netted control plots, I = un-netted plots treated with insecticides).

Target	Applied on	Active Ingredient	Trade Name	Year	No. of Treatments
*Grapholita molesta*	I	Chlorpyrifos methyl	Reldan^TM^	2016	1
	I	Etofenprox	Trebon^®^ UP	2016	1
	I	Phosmet	Spada^®^	2017	1
	I	Chlorpyrifos methyl	Pyrinex^®^	2017	2^▲^
	I	Etofenprox	Trebon^®^ UP	2017	2
	I	Chlorpyrifos methyl	Runner^®^ LO	2017	1
*Halyomorpha halys*	I	Deltamethrin	Decis^®^	2017	1
*Monilia* spp.	N, C, I	Sulfur	Tiovit^®^	2016	1
	N, C, I	Tebuconazole, sulfur	Tebusip^®^	2016	2
	N, C, I	Tebuconazole, sulfur	Tebusip^®^	2017	1
	N, C, I	Tebuconazole	Dedalus^®^ 25 WDG	2017	1
*Monilia* spp., *Podosphaera pannosa*	N, C, I	Sulfur	Tiovit^®^	2017	2

^▲^ Insecticide treatment applied to the alternate row.

**Table 2:** Cumulative number of *Grapholita molesta* per trap (mean ± SE) and percentages of nectarines damaged by *G. molesta* (mean ± SE) on fruits sampled at harvest (N = netted plots, C = un-netted control plots, I = un-netted plots treated with insecticides). For each cultivar and year, means followed by different letters are significantly different (traps: *t*-test, *p* < 0.05; damaged fruits: generalized linear mixed model (GLMM) analysis, Bonferroni correction, *p* < 0.05).

Cultivar	Treatment	*Grapholita molesta* (no.)		Damaged Nectarines (%)	
		2016	2017	2016	2017
Amiga*	N	5.0 ± 2.1 b	39.0 ± 9.2 b	0.4 ± 0.2	0.6 ± 0.3 c
	C	22.7 ± 1.7 a	160.3 ± 11.5 a	4.2 ± 0.8	9.4 ± 0.9 a
	I	-	-	2.1 ± 0.5	3.4 ± 0.6 b
Fire Top^®^	N	8.7 ± 3.3 b	39.0 ± 4.6 b	0.0 ± 0.0	0.4 ± 0.2 b
	C	38.3 ± 1.9 a	174.7 ± 30.4 a	0.1 ± 0.1	6.2 ± 0.9 a
	I	-	-	0.1 ± 0.1	4.1 ± 0.7 a

**Table 3:** Cumulative number of *Drosophila suzukii* (mean ± SE) and predators (mean ± SE) collected per trap in the nectarine orchards (N = netted plots, C = un-netted control plots). For each cultivar and year, means followed by different letters are significantly different (*t*-test, *p* < 0.05).

Cultivar	Treatment	*Drosophila suzukii* (no.)		Predators (no.)	
		2016	2017	2016	2017
Amiga*	N	7.0 ± 1.0 b	7.0 ± 2.3 b	1.7 ± 1.2 b	7.7 ± 2.0 b
	C	60.7 ± 3.4 a	21.3 ± 1.8 a	41.0 ± 3.0 a	74.0 ± 2.0 a
Fire Top^®^	N	13.0 ± 7.6 b	12.0 ± 2.5 b	1.0 ± 0.6 b	1.3 ± 0.3 b
	C	129.0 ± 25.2 a	33.7 ± 6.4 a	25.7 ± 1.7 a	58.7 ± 9.6 a

**Table 4:** Percentages of postharvest rots (mean ± SE) on nectarines after storage and after shelf life. Fruits were harvested in 2016 and in 2017 (no. = 900 fruits per treatment per year) (N = netted plots, C = un-netted control plots, I = un-netted plots treated with insecticides). For each cultivar and year, means followed by different letters are significantly different (Tukey’s Test, *p* < 0.05).

Cultivar	Treatment	Postharvest Rots Incidence (%)			
		After Storage		After Shelf Life	
		2016	2017	2016	2017
Amiga*	N	0.4 ± 0.3	0.0 ± 0.0	4.9 ± 1.6 b	4.2 ± 1.9 b
	C	0.4 ± 0.5	0.6 ± 1.0	11.0 ± 4.2 a	14.3 ± 2.5 a
	I	0.0 ± 0.0	0.0 ± 0.0	3.3 ± 1.8 b	6.8 ± 4.4 ab
Fire Top^®^	N	0.7 ± 0.9	2.2 ± 1.3 b	15.9 ± 7.2 b	14.1 ± 8.9 b
	C	4.0 ± 1.8	0.7 ± 1.3 b	28.6 ± 8.4 a	37.6 ± 2.3 a
	I	1.6 ± 0.9	10.0 ± 4.8 a	26.7 ± 11.3 a	33.6 ± 15.2 ab

**Table 5:** Color index, firmness, total soluble solids, total polyphenols and total anthocyanins (mean ± SE) of the nectarines harvested in 2017 (N = netted plots, C = un-netted control plots, I = un-netted plots treated with insecticides). In each column for each cultivar, means followed by different letters are significantly different (Amiga* total solid soluble and Fire Top^®^ color index: Tukey’s test, *p* < 0.05; Fire Top^®^ total solid soluble: Mann–Whitney U test, *p* < 0.05).

Cultivar	Treatment	Color Index	Firmness (g cm^−2^)	Total Soluble Solids (°Brix)	Total Polyphenols (mgGAE 100 g^−1^)	Total Anthocyanins (mgC3G 100 g^−1^)
Amiga*	N	30.8 ± 1.0	4.9 ± 0.1	8.2 ± 0.1 b	33.8 ± 2.4	7.4 ± 2.0
	C	30.8 ± 1.2	4.9 ± 0.1	8.6 ± 0.1 a	31.1 ± 1.9	12.3 ± 1.8
	I	27.8 ± 1.1	5.2 ± 0.1	8.4 ± 0.1 ab	29.0 ± 2.3	9.5 ± 2.2
Fire Top^®^	N	36.7 ± 1.9 b	4.2 ± 0.1	8.3 ± 0.1 c	65.4 ± 6.9	9.7 ± 2.0
	C	44.9 ± 1.6 a	4.2 ± 0.1	8.7 ± 0.1 b	51.5 ± 3.7	11.0 ± 2.3
	I	38.0 ± 1.6 b	4.1 ± 0.1	8.9 ± 0.1 a	47.9 ± 2.0	13.3 ± 2.6

## Data Availability

Data is contained within the article.
